# Supplementary material for: Energy consumption and cooperation for optimal sensing
Source: Nat Commun. 2020 Feb 20;11:975. doi: 10.1038/s41467-020-14806-y (PMC7033096; doi:10.1038/s41467-020-14806-y)
Supplement: Supplementary file 1 — Supplementary Information [file 41467_2020_14806_MOESM1_ESM.pdf]

Energy consumption and cooperation for optimal sensing: Supplementary Information

Vudtiwat Ngampruetikorn,[1,2] David J. Schwab,[2,3] and Greg J. Stephens[4,5]

[1] *Department of Physics and Astronomy, Northwestern University, Evanston, IL 60208 USA*
[2] *Initiative for the Theoretical Sciences, The Graduate Center, CUNY, New York, NY 10016 USA*
[3] *Center for the Physics of Biological Function, Princeton/CUNY*
[4] *Biological Physics Theory Unit, OIST Graduate University, Okinawa 904-0495, Japan*
[5] *Department of Physics and Astronomy, Vrije Universiteit, 1081HV Amsterdam, The Netherlands*

(Dated: February 3, 2020)

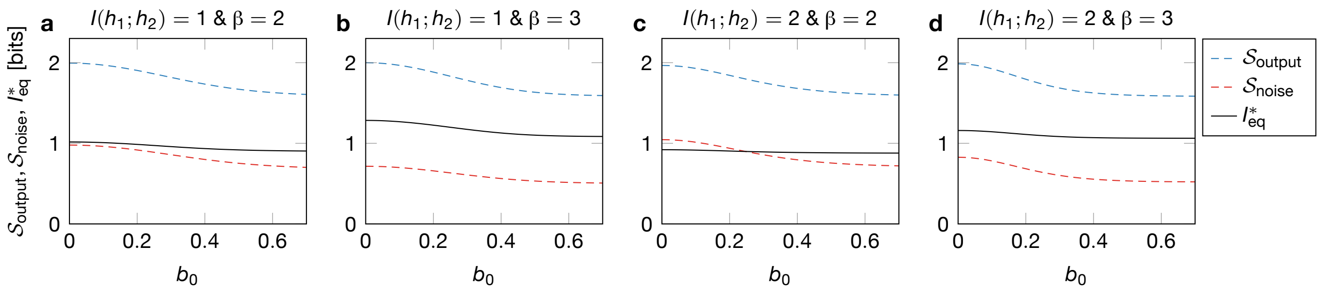

Supplementary Figure 1. Intrinsic (signal-independent) sensor biases *decrease* mutual information in optimal equilibrium sensing. We subject two sensors to bivariate Gaussian signals [Eq. (10)] and a signal-independent bias b_0 such that the net bias becomes $(h_1 + b_0, h_2 - b_0)$. We then optimise the sensor coupling J to obtain maximum mutual information and the corresponding noise and output entropies (a-d). We depict the effects of intrinsic biases for four combinations of signal redundancy $I(h_1; h_2)$ and sensor reliability β (see panel labels), which are representative of the cases where the optimal sensing strategy is equilibrium (a,b) and nonequilibrium (c,d) (see also, Fig. 2**b**).

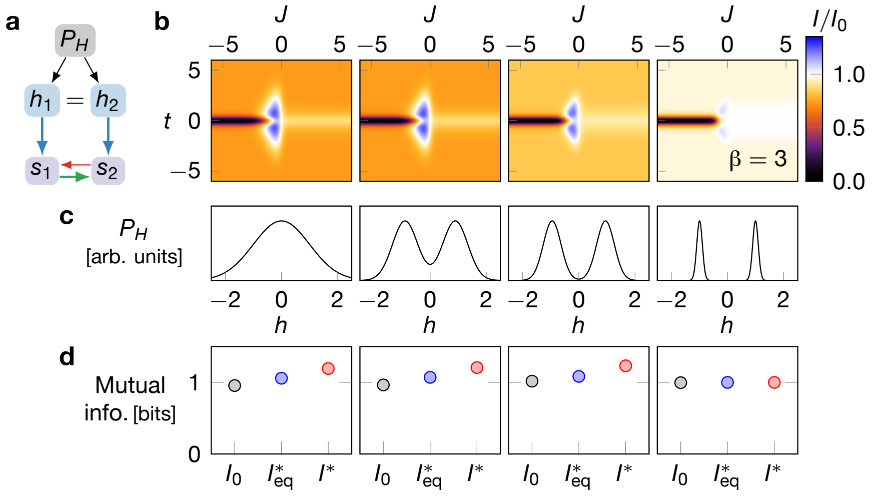

Supplementary Figure 2. For highly correlated signals, the nonequilibrium improvement in the low-noise limit does not rely on the specific Gaussian prior. (**a**) We assume that two sensors are driven by the same bias field $h \equiv h_1 = h_2$. (**b**) The mutual information at sensor reliability of $\beta = 3$ for four different prior distributions with a zero mean and unit variance, shown in **c**. For all priors considered, the optimal nonequilibrium drive t^* is clearly finite, suggesting that the nonequilibrium enhancement is robust for most continuous priors. The dependence of the mutual information on J and t remains qualitatively unchanged. This means the mechanism behind the nonequilibrium enhancement is likely to be identical to the one for a Gaussian prior, as described in the main text. (**d**) The maximum mutual information — I_0, $I^*_{\rm eq}$ and I^* — for noninteracting, equilibrium and nonequilibrium sensors, respectively. The nonequilibrium enhancement is visible in all cases except for the most binary-like one (far right).

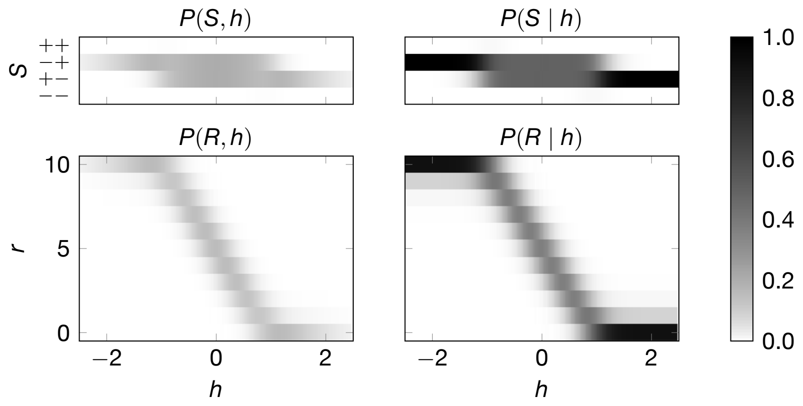

Supplementary Figure 3. The ladder structure in Fig. 5**e** corresponds to the anticorrelated sensor states $+-$ and $-+$. The joint (left) and conditional (right) probability distributions of the sensor states (top) and the readout population (bottom). Here we use, as in Fig. 5**e**, $J = -2$, $t = 7$, $\delta = -0.6$, $\Delta = 1$ and $r_0 = 10$.

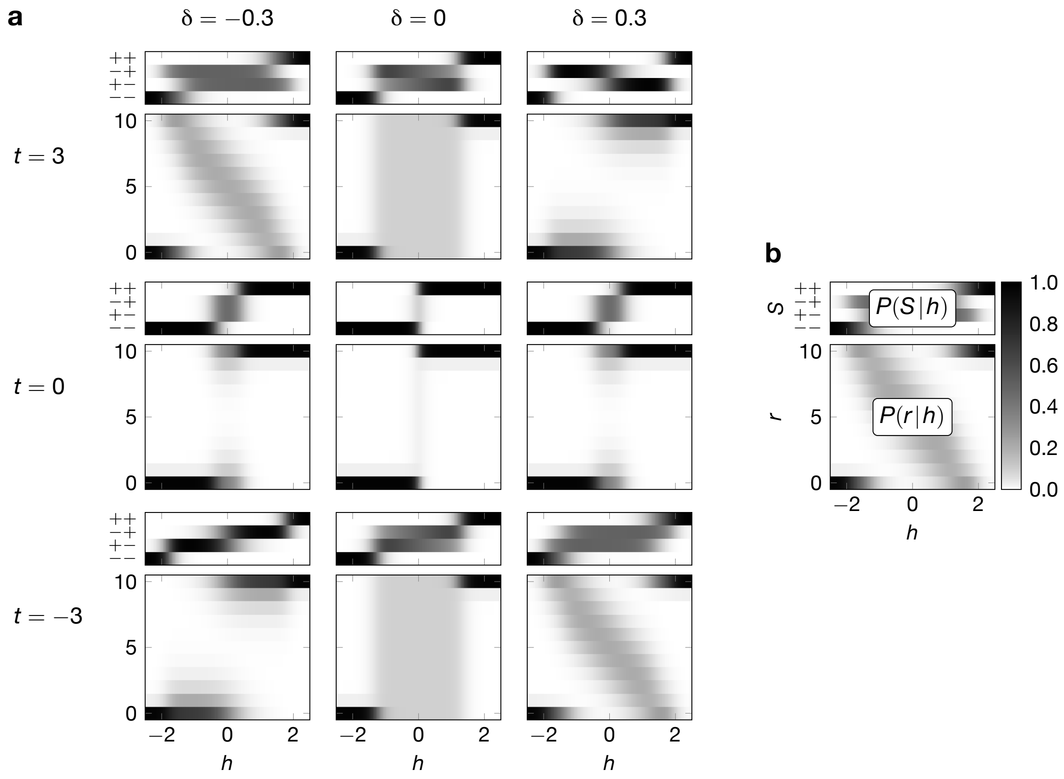

Supplementary Figure 4. (**a**) Conditional probabilities of sensor state and readout given a perfectly correlated signal (see, (b) for legend) at $J = -2$ and $\beta = 4$ for $t = 3, 0, -3$ (top-to-bottom) and $\delta = -0.3, 0, 0.3$ (left-to-right). A strong signal $|h| \gg 1$ favours the correlated sensor states ($++$ and $--$) and the extreme readout states ($r = r_0$ and $r = 0$), a finite nonequilibrium drive $t \neq 0$ lifts the degeneracy between the sensor states $-+$ and $+-$ (cf. Fig. 4), and $\delta \neq 0$ differentiates the intermediate readout states ($0 < r < r_0$). The ladder structure in Fig. 5**e** requires both t and δ to be non-zero. Here we use $\Delta = 1$ and $r_0 = 10$.
